# Associations of sleep time, quality of life, and obesity indicators on physical literacy components: a structural equation model

**DOI:** 10.1186/s12887-024-04609-1

**Published:** 2024-03-07

**Authors:** Vanilson Batista Lemes, Ana Paula Sehn, Cézane Priscila Reuter, Ryan Donald Burns, Anelise Reis Gaya, Adroaldo Cesar Araujo Gaya, Caroline Brand

**Affiliations:** 1 Federal University of Rio Grande do Sul, Porto Alegre, Brazil; 2 University of Santa Cruz do Sul, Santa Cruz do Sul, Brazil; 3 University of Utah, Salt Lake City, USA; 4 IRyS Group, Physical Education School, Pontificia Universidad Católica de Valparaíso, Valparaíso, Chile

**Keywords:** Physical education, Physical activity, Fitness, Adolescents

## Abstract

**Aim:**

To verify the association between ideal sleep time and physical literacy components while also considering multiple mediators, such as quality of life and obesity, using a sample of adolescents.

**Methods:**

This cross-sectional study consisted of 470 adolescents aged 11–17 years from southern Brazil. Sleep time, health-related quality of life, and physical literacy components (i.e., physical education enjoyment, sports participation, sedentary behavior, moderate to vigorous physical activity, sex, and age) were evaluated through self-reported questionnaires. In addition, body mass index (BMI), and waist circumference were determined. The theoretical/statistical support of the structural equation model was evaluated according to fit parameters and strength of association.

**Results:**

A direct association was observed between more sleep time and lower levels of obesity. The obesity indicators also had a negative association with HqOL, and HqOL had a positive association with physical literacy. The indirect associations indicated that the ideal sleep time was positively associated with HqOL and physical literacy components, considering the negative mediation effect of obesity. The model explains physical literacy in 31% of the variance (*R* = 0.31).

**Conclusion:**

There was an indirect association between ideal sleep duration and quality of life and between both variables with physical literacy. These relationships occur even considering the negative influence of obesity. Therefore, a child who sleeps adequately has a higher likelihood of being physically active, regardless of obesity, potentially enhancing overall quality of life across various domains.

## Introduction

Adequate sleep plays a crucial role in maintaining optimal health and well-being in children and adolescents [1]. During sleep, children’s bodies undergo essential processes to repair and regenerate tissues, support immune function, and consolidate newly acquired knowledge and skills [2].

Despite recommended guidelines suggesting that adolescents should sleep 8 to 10 h regularly to promote optimal health [3], a systematic review incorporating data from multiple countries, including Brazil, revealed that children and adolescents sleep less than the recommended amount [4]. As a consequence of insufficient sleep, these individuals present an increased risk of negative outcomes, such as the increased risk of obesity, behavioral problems [5], and impaired neurocognitive development [6].

Conversely, regular physical activity is associated with numerous health benefits, including improved sleep quality, enhanced quality of life, increased physical fitness, and a reduction of obesity and cardiometabolic risk factors [7, 8]. Recognizing the importance of both sleep and physical activity f or overall health, it is essential to address these factors within the concept of physical literacy. Physical literacy encompasses a range of dimensions, including affective factors such as motivation and confidence, physical competence, movement, physical fitness, cognitive aspects such as knowledge and understanding, and behavioral elements related to lifelong engagement in physical activities [9]. By integrating these dimensions, physical literacy offers a complex perspective on physical activity that recognizes the significance of social processes in lifelong learning [10]. As physical activity levels decline and sedentary behavior rises, physical literacy has emerged as a valuable strategy to counteract these challenges [11]. By promoting physical literacy, children and adolescents can develop the necessary skills, knowledge, and motivation to actively participate in physical activities throughout their lives, consequently promoting healthier lifestyles [12].

However, there are limited studies specifically examining the influence of sleep on physical literacy, although some evidence highlights its impact on the individual components of physical literacy [12–14]. Sleep plays a vital role in affecting factors such as physical activity enjoyment and improved sports performance, as individuals who are well-rested tend to have higher levels of motivation and engagement in physical activities [15]. Additionally, adequate sleep contributes to reduced sedentary behavior, as individuals who experience quality sleep and appropriate sleep time are more likely to engage in active pursuits and limit sedentary time [16, 17].

Therefore, the present study proposes a comprehensive and complex model to approach the multiple relationships and influences on the construct of physical literacy. In this context, we hypothesized that quality of life and obesity indicators could potentially mediate the association between sleep and physical literacy. This hypothesis is supported by existing evidence indicating the interplay between these factors. The literature has suggested that adolescents with poor sleep quality and shorter sleep duration present lower health-related quality of life [18, 19]. Furthermore, obesity indicators, such as body mass index (BMI) and waist circumference, also seem to influence sleep and physical literacy. However, these associations remain uncertain and require further confirmation [20]. As mentioned earlier, sleep deprivation and poor sleep quality have been associated with an increased risk of obesity [21]. Obesity, in turn, can impact physical literacy by affecting physical competence, motivation, and self-confidence, which are essential dimensions of physical literacy [20]. The mechanisms linking sleep to enhanced physical literacy remain unclear. However, the present study speculates that increased sleep duration may contribute to greater engagement in physical activity and sports [22], both fundamental aspects of physical literacy. Furthermore, correlations may exist between sleep, physical literacy, and various factors such as physical and mental well-being, quality of life, and cognition. This research aims to address the existing gap in the literature surrounding this topic.

Thus, the aim of the present study was to verify the association between ideal sleep time and physical literacy components considering the multiple mediators’ relationship among quality of life and obesity indicators in adolescents.

## Methods

### Study design, sample and ethical procedures

A cross-sectional study with a quantitative approach was conducted according to previous published work [17, 23–25].

The study population consisted of 1570 adolescents from seven state elementary schools located in a city in Rio Grande do Sul, Brazil. Four schools were selected using convenience criteria, which included factors such as the highest number of enrollments in the state elementary school system in the city, with approximately 1166 students, accounting for 74% of the total population of the seven schools. The selection also considered variations in educational development indexes (IDEB) among the schools and their distinct geographical distribution across the four regions of the city: north-center, south-center, east, and west [17, 23–25].

The sample size (N) estimated to represent the population of students consisted of a minimum of 470 subjects (accounting for a 20% allowance for potential losses). This sample size was obtained using G*Power version 3.1 software, considering a multivariate test of associations involving 15 to 20 predictor variables, test power of 0.80, alpha of 0.05, and an effect size of 0.10 (r) [16, 17, 26, 27]. The mathematical assumption of at least 20 subjects for each endogenous or exogenous variable included in the structural equation model (SEM) was also considered [28].

Participants were selected from the sixth to ninth grades of elementary school in 2017, encompassing adolescents aged 11 to 17 years (Mean: 13.22 ± 1.50). The distribution of participants across schools was determined based on the number of enrollments in each respective class, and the selection of subjects was made through a randomization process [17, 24, 27, 29]. According to the enrollment proportions at each institution, a total of 470 participants were included in the present sample. The distribution of participants across the schools is as follows: School 1, *N* = 135; School 2, *N* = 139; School 3, *N* = 51; and School 4, *N* = 145 [17, 24, 27, 29].

The study was approved by the ethics and research committee of the Federal University of Rio Grande do Sul, approval number: 3.634.294. The research followed ethical guidelines for procedures involving human subjects in accordance with the Helsinki Declaration. Adolescents were included in the study with their assent and informed, free, and clear consent from their guardians.

### Measured variables

#### Procedures and bias risk minimization

Two teachers (one man and one woman) applied the evaluations separately to girls and boys. Possessing 5 years of experience in Physical Education and research in human movement sciences, they were specialists in physical assessment and anthropometry. Additionally, training sessions were conducted encompassing all variables involved in the current study. Initially, data were documented on paper sheets and subsequently transcribed into Excel for analysis.

Concerning evaluations, a class period of 45 to 60 min was used for administering the questionnaire in each class with approximately 15 to 30 subjects. We requested complete sincerity and instructed the adolescents not to look at the answers of other participants, assuring them not to be concerned about it. They were informed about the importance and relevance of providing accurate and realistic data for scientific research. We provided examples to explain physical activities in general, such as physical education classes, sports, daily activities, and exercise. Emphasis was placed on explaining the difference between moderate and vigorous physical activities and light physical activities.

Using the classroom blackboard, we demonstrated through examples how to measure minutes of physical activity or sedentary behavior during the morning, afternoon, and evening. Participants were encouraged to recall activities they typically engaged in or places they usually frequented during each time period, facilitating their recollection.

Students were assisted in answering the first question and calculating the minutes of physical activity, sleep, and sedentary behavior during the questionnaire administration. Students were allowed to use calculators and electronic devices to optimize calculations. Whenever students requested assistance, we provided situational examples to clarify the questions.

#### Ideal sleep time (independent factor)

Sleep Time (main independent observed variable in SEM) was evaluated according to Movement Behavior Questionnaire (MBQ) (Cronbach/Omega reliability = 0.64 to 0.94) proposed by Lemes et al. [30] considering the question: (On average, how many hours do you sleep per night?). In the present study, we classified the Ideal Sleep time according to specific guidelines; It was ranged between 8 and 11 h by night [3, 27].

#### Health-related quality of life (HqOL - latent mediator variable)

HqOL was evaluated using a scoring system based on the Kidscreen-27 questionnaire, which has been validated and translated into Brazilian Portuguese for children and adolescents aged 10 to 18 years [17, 23]. In the present study, the self-reported HqOL latent variable encompassed the 27 questions organized with an ordinal scale composed of 1–4 and 1–5 levels. The domains of HqOL are physical well-being (5 items); psychological well-being (7 items); autonomy and parents (7 items); social support and peers (4 items), and school environment (4 items). In the present study, all questions are used in the same latent construct. These can be observed as follows: 1-In general. How is your health? 2-Have you been feeling good and willing? 3-Have you been practicing physical activities? 4-Have you been able to run well? 5-Have you been feeling energetic? 6-Has your life been pleasant? 7-Have you been in a good mood? 8-Have you been having fun? 9-Have you been feeling sad? 10-Have you been feeling so bad that you didn’t feel like doing anything? 11-Have you been feeling alone?12-Do you feel happy the way you are?13-Have you had enough time for yourself?14-Have you been doing the things you want in your spare time?15-Do your parents have enough time for you?16-Do your parents treat you fairly?17-Are your parents available to speak when you want to?18- Do you have enough money to do the same things as your friends?19- Do you have enough money for your expenses? 20-Have you been spending time with your friends? 21- Do you have fun with your friends? 22- Do you and your friends help each other? 23-Do you trust your friends? 24-Do you feel happy at school? 25-Are you doing well at school? 26-Have you been able to pay attention at school? 27-Do you get along well with your teachers?

#### Obesity indicators (latent mediator variable)

Height was measured in centimeters (cm) using a metal measuring tape (Cescorf) fixed on the wall at a distance of 1 m from the ground and extended from bottom to top; body mass was measured using a Digital portable scale (OMRON), with a precision of up to 100 g [27, 31]. The device was calibrated according to the manufacturer’s specifications to ensure accuracy and reliability. This process involved placing a known weight on the Omron HBF-214 digital scale. The calibration button was then pressed until the display accurately reflected the weight value. Following this, the device was turned off and then turned back on to complete the calibration process. The students were assessed with minimal clothing, preferably barefoot. The measurement was obtained in kilograms (kg). The calculation of BMI was performed by dividing body mass by height (in meters) squared. The BMI was classified into risk zone and healthy zone according to the criteria of the Sport Brazil Project (PROESP-BR) for adolescents aged 11 to 17 years. Based on this classification, the occurrence of children in the health risk or healthy zone was calculated for the variable [27, 31]. The waist circumference (WC) was measured to the nearest millimeter (mm) using a metallic tape (Brand Cescorf) at the upper border of the iliac crest [24]. Four measurements were taken, and the one that was repeated twice was considered.

#### Physical literacy components for a math construct (latent dependent variable)

The Physical Literacy components are a specific construct with variables possible for evaluation in the context of the present study. It was composed of movement, enjoyment, and personal factors associated with physical activity practice over of lifetime [10]. Following this theoretical concept, sex and age were evaluated according to self-description in questionnaires. In addition, physical education enjoyment, sports participation, moderate to vigorous physical activity (MVPA) time in one day, and sedentary behavior time at school in one day were evaluated according to the procedures described below. Therefore, the dimensions of physical literacy considered in the present study includes physical activity enjoyment, sedentary behavior (as a negative factor), MVPA, sports practice, sex, and age.

#### Physical education enjoyment (PE enjoy)

It was measured using a scoring system based on a previous instrument validated in the Brazilian context [17, 27, 32]. The instrument assesses whether adolescents feel good, have their psychological needs met, and experience positive feelings during physical education. In the present study, four domains of the questionnaire were used: 1-Perceiving physical education classes as interesting; 2-Perceiving physical education as fun; 3-Engaging in physical education; 4-Liking physical education.

#### Sports participation

Sports practice was assessed considering that the sample consisted of adolescents aged 11 to 17 years in a learning phase, using the following question: In the last 7 days, did you participate in or engage in any sports activities involving body movement? (Response options: yes or no) [17, 23].

#### Sedentary time and levels of moderate to vigorous physical activity

These variables were also measured using the MBQ for Brazilian schoolchildren [33]. The questionnaire consists of nine questions regarding MVPA levels on weekdays and the duration in minutes during the morning, afternoon, and evening [17, 24, 30]. The sedentary time in the morning, afternoon, and evening was also evaluated. The periods of the day are summed to obtain daily physical activity and sedentary time in one day in minutes. In the present study, the MVPA sufficient was classified according to Canadian Guidelines: 60 min or more is a sufficient time and above is an insufficient health MVPA in one day. Sedentary behavior out of school is the time in minutes per day minus the period in the school (240 min) [17, 24, 30].

#### Statistical analysis

We conducted a descriptive analysis of the data in IBM SPSS and AMOS graphics, presenting means, standard error, occurrences, absolute values, and confidence intervals for a bootstrapping with 1000 repetitions. Prior to performing the mathematical model, we conducted a preliminary analysis to assess the multivariate normality of the data and identify any potential outliers using the Mahalanobis distance centroid test. The results indicated that it is feasible to apply a structural equation model (SEM) to the present dataset. Furthermore, we considered similar studies in the field to ensure the robustness of our approach [17, 34].

#### Structural equation model with a mediation conception

**Fig. 1:**
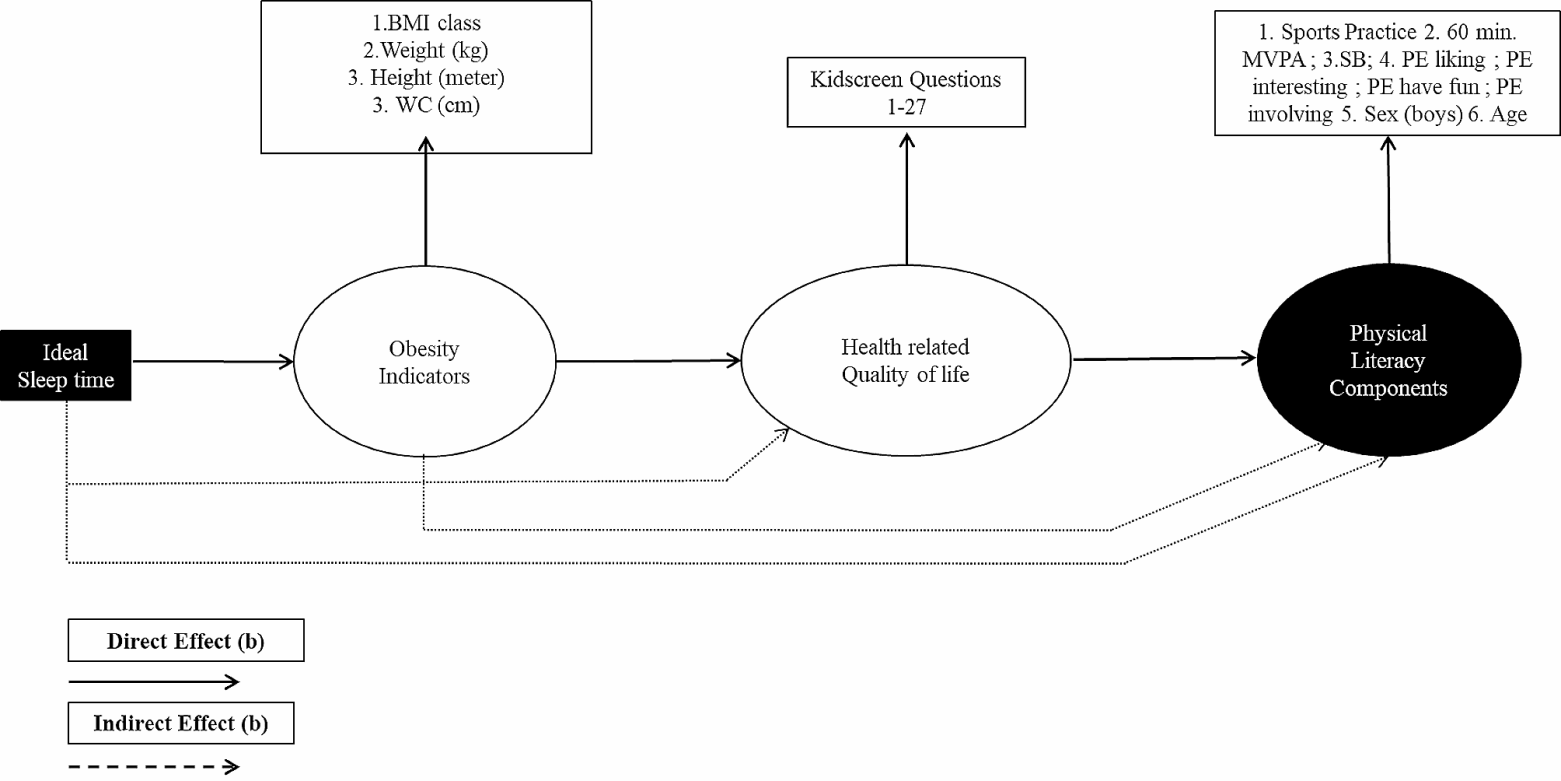
Proposal for possible mediators in the association between ideal sleep time and physical literacy components; BMI. Body mass index; WC. Waist circumference; MVPA. Moderate to vigorous physical activity; SB. Sedentary behavior; PE liking = liking physical education; PE interesting = perceiving physical education classes as interesting; PE have fun. Perceiving physical education as fun; PE involving = Engaging in physical education

The proposed structural equation model (Fig. 1) was developed based on the objectives of the present study: to verify the association between ideal sleep time and physical literacy components considering the multiple mediator’s relationship among quality of life and obesity indicators in adolescents. Considering previously presented approaches [17, 34]. The new view in this model is recursive with an endogenous (dependent) main variable that is PL. In this model, all variables can contribute directly or indirectly to multiple relationships among themselves, considering the impact of association from the left to the right side, conform the proposal at Fig. 1. All model directs and indirect effects were calculated considering a bootstrapping for 5.000 samples [35].

The relationship among variables was estimated according standardized β values, indirect and direct relationships were observed. The coefficient of determination was calculated for each one of indicators to latent variable as well, the factorial charge (β). To assess the goodness of fit for this model, several fit indices were examined. These indices include Standardized Root Mean Square Residual (SRMR): The SRMR should be less than 0.10, Chi-Square divided by degrees of freedom (CMIN/DF) This should be less than 5; Root Mean Square Error of Approximation (RMSEA), it should be less than 0.077 (0.074–0.080; p-close: 0.001), indicating a good fit [17, 23, 34, 35]. Also, the significance level was set as *p* < 0.05.

## Results

The sample characteristics are present in Table 1. The findings revealed that a significant proportion of adolescents had inadequate sleep time (77.40%), were classified in the risk zone for BMI (63.80%), had insufficient MVPA levels (36.80%), and reported being engaged in sports practice (69.80%).

**Table 1:** Description of sample variables

Nominal/Ordinal Variables		Frequency	Percent	Std. Error	Bootstraping CI (95%)	
Sex	Girls	240	51.10	2.30	46.60	55.50
	Boys	230	48.90	2.30	44.50	53.40
Age (Years)	11	55	11.70	1.50	8.70	14.70
	12	117	24.90	1.90	21.30	28.90
	13	107	22.80	1.90	19.40	26.60
	14	99	21.10	1.90	17.20	24.50
	15	54	11.50	1.40	8.90	14.50
	16	26	5.50	1.10	3.60	7.90
	17	12	2.60	0.70	1.30	4.00
Sleep Time	Inadequate	364	77.40	1.90	73.80	81.10
	Ideal	106	22.60	1.90	18.90	26.20
BMI (Proesp-Br)	Healthy	300	63.80	2.20	59.60	68.30
	Risk	170	36.20	220	31.70	40.40
MVPA	Insuficient	173	36.80	2.20	32.30	41.50
	> 60 min	297	63.20	2.20	58.50	67.70
Sports Practice	No	142	30.20	2.10	26.20	34.30
	Yes	328	69.80	2.10	65.70	73.80
Physical Education Enjoyment and HqOL (Level 4 and 5 Classification)	Enjoy (Interesting)	150	31.90	2.20	27.90	36.60
	Enjoy (Having fun)	192	40.90	2.30	36.60	45.50
	Enjoy (Involving)	155	33.00	2.20	28.90	37.40
	Enjoy (Liking)	204	43.40	2.30	39.10	48.10
	HqOL1	77	16.40	1.70	13.20	19.80
	HqOL2	68	14.50	1.60	11.30	17.70
	HqOL3	103	21.90	1.90	18.10	25.50
	HqOL4	74	15.70	1.70	12.60	19.10
	HqOL5	107	22.80	2.00	18.90	27.00
	HqOL6	121	25.70	1.90	22.10	29.60
	HqOL7	115	24.50	2.00	20.60	28.70
	HqOL8	185	39.40	2.30	34.90	43.40
	HqOL9	14	3.00	0.80	1.70	4.70
	HqOL10	20	4.30	0.90	2.60	6.20
	HqOL11	14	3.00	0.80	1.70	4.50
	HqOL12	240	51.10	2.30	46.60	56.00
	HqOL13	150	31.90	2.10	27.90	36.20
	HqOL14	134	28.50	2.10	24.50	33.00
	HqOL15	178	37.90	2.20	33.60	42.30
	HqOL16	184	39.10	2.20	34.70	43.60
	HqOL17	204	43.40	2.30	38.90	47.90
	HqOL18	105	22.30	2.00	18.50	26.20
	HqOL19	162	34.50	2.20	29.80	38.90
	HqOL20	200	42.60	2.30	37.90	47.00
	HqOL21	300	63.80	2.30	59.40	68.50
	HqOL22	225	47.90	2.30	43.40	52.30
	HqOL23	207	44.00	2.30	39.40	48.30
	HqOL24	57	12.10	1.50	9.10	15.10
	HqOL25	68	14.50	1.60	11.30	17.70
	HqOL26	136	28.90	2.10	24.90	33.20
	HqOL27	195	41.50	2.20	36.80	45.70
Continuous variables			Mean	Std. Error	CI (95%)	
	Height (meters)		1.61	0.01	1.60	1.62
	Weight (kg)		56.84	0.73	55.46	58.28
	Waist Circumference (cm)		70.82	0.49	69.88	71.79
	Out of School SB (minutes)		401.79	9.72	383.19	421.63

HqOL. Health related quality of life; MVPA. Moderate to vigorous physical activity; SB. Sedentary behavior; CI. Confidence interval

The goodness fit of the model for the data basis presents adequate parameters for a mediation analysis, considering our previous definition. A SRMR = 0.0791, CMIN/DF = 3.786 and the RMSEA = 0.077.

**Fig. 2:**
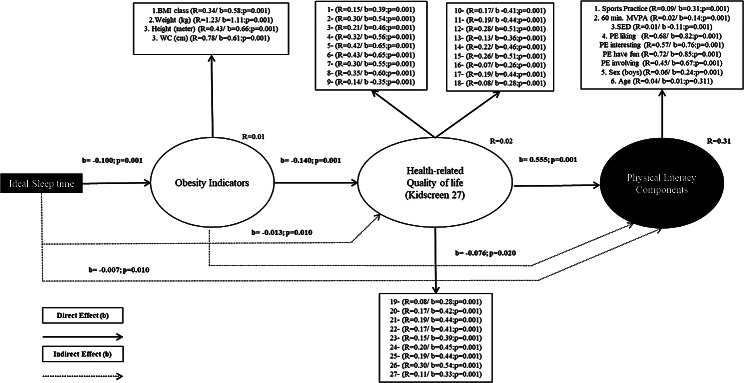
Structural Equation Model for ideal sleep time and physical literacy components considering the multiple mediator’s relationship among quality of life and obesity indicators in adolescents. Number 1–27 in health-related quality of life latent construct are the questions from Kidscreen; MVPA. moderate to vigorous physical activity; SB. sedentary behavior out of school; PE: physical education enjoyment domains; BMI. body mass index; WC. waist circumference; R. determination coefficient, multiple correlations squared; b. beta correlation effect; p. signifance level less than 0.05

The main direct and (mediated) indirect relationships in the model are presented in Fig. 2. The coefficients (b) in the model are measured using to z-score, and the significance level is shown as well. Additionally, the determinant coefficient (R^2^) is presented to assess the overall fit of the model.

According to Fig. 2 it is possible to perceive the direct association between more sleep time and less obesity indicators levels. However, the obesity indicator levels have a negative association with HqOL, and HqOL has a positive association with PL components in the latent construct.

The indirect associations indicate that the ideal sleep time was positively associated with HqOL and physical literacy components, even considering the negative mediation power of obesity indicators. Which, in turn, has an indirect negative and significant association with PL mediated by HqOL.

In the model, physical literacy components are collectively explained by ideal sleep time, obesity indicators and HqOL, accounting for 31% of the variance (R^2^ = 0.31). Also, the observed variables comprising the latent constructs demonstrate a satisfactory and statistically significant relationship with the model. The only variable that does not present a significative relation with the model is Age (R^2^ = 0.04/b = 0.01; *p* > 0.31).

## Discussion

The findings of the present study suggested that achieving an ideal sleep time is positively associated with components of PL. This association was mediated by an improvement in the HqOL. However, it is important to note that the presence of obesity indicators negatively influenced these relationships, creating obstacles to achieving ideal sleep, HQOL, and physical literacy components. Nevertheless, our structural equation model (SEM) demonstrated satisfactory goodness of fit. Overall, the model accounted for 31% of the variance in the physical literacy construct. We emphasize the significant findings of the present study as it considers the interrelation between various lifestyle behaviors, recognizing the pivotal importance of sleep duration. This allowed us to obtain an enhanced understanding of how children manage to balance periods of sedentary behavior and physical activity, and how these factors directly and indirectly impact obesity and health-related quality of life (HQOL).

Sleep is an important factor associated with health and well-being [36, 37]. Evidence from the literature indicated that adequate sleep duration and quality seem to influence physical literacy components, such as pleasure in physical activity, MVPA, playing sports, and sedentary behavior. The authors of the present study present the hypothesis that ideal sleep is important due to the fact that by presenting an ideal quality and quantity of sleep, people have a better mood and willingness to carry out activities throughout the day, facilitating participation in physical activities and reducing sedentary behavior. In this sense, a study concluded that the presence of smartphones during sleep hours is detrimental to ideal sleep due to time spent in front of smartphones being associated with short sleep duration, reflecting negatively on daily functioning and mood [38]. Furthermore, other factors can influence the sleep patterns of adolescents, such as high social involvement, frequent attendance at parties, study shifts, increased time spent in sedentary behaviors, and entry into the workforce [2, 39]. A systematic review study with meta-analysis pointed out an association between longer sleep duration, lower sedentary behavior, and higher physical activity during the day [36]. Another study observed that sleeping early is associated with higher MVPA time and higher sedentary behavior. Also, it was suggested that sleep duration, physical activity, and sedentary behavior presented complex, bidirectional, and personal associations [40]. In addition, improving sleep health is essential to reducing screen time, and it is suggested that perceived sleep quality seems to promote healthy behaviors the following day [41].

HQOL also seems to be benefited from ideal sleep. It was indicated that adequate sleep duration is positively associated with better HQOL [42], including in Brazilian young [37]. Other study pointed out that sleep duration is predicted of mental well-being [43]. In children, it is also observed the relationship between ideal sleep and better HQOL [44]. A systematic review found that adolescents with more hours of sleep had better HQOL and lower adiposity compared with individuals with short sleep duration [45]. Regarding obesity indicators, results of a systematic review study suggested that there is an association between adequate sleep duration and the reduction of weight in children and adolescents and vice versa [46]. Sleep disorders seem to be a common complication of being overweight, and sleep disorders are also capable of worse complications associated with adiposity [47]. A meta-analysis identified that short sleep duration is longitudinally associated with childhood obesity [48]. However, it is important to highlight that sleep presents U-shaped patterns, both short-duration and long-duration sleep (> 9 h) demonstrate negative health effects [49, 50]. Therefore, adhering to the recommended sleep duration of 8 to 10 h is essential to promote optimal health in adolescents [3].

However, the findings of the present study indicate that obesity has a detrimental impact on the relationships between ideal sleep, health-related quality of life (HQOL), and physical literacy. This outcome raises concerns due to the multifactorial nature of obesity, highlighting the need to address various factors in order to effectively reduce childhood obesity. Consequently, only meeting sleep recommendations is not considered a sufficient strategy for reducing obesity [48]. The existing literature provides further evidence supporting the association between being overweight and experiencing poorer health-related quality of life (HQOL), as well as reduced levels of physical literacy components [51], such as physical activity practice and sedentary behavior [52, 53]. Additionally, studies have shown that the inverse relationship between sleep duration and adiposity in adolescents also impacts HQOL and physical literacy [51].

Therefore, present findings have indicated that sleep is fundamental to several health indicators. However, the physical literacy components considered in the present study, especially practicing regular physical activity, physical exercise, and sedentary behaviors also seem to develop beneficial effects to reduce obesity and improve HQOL in adolescents. It is highlighted that strategics of public health should encourage children and adolescents at early bedtime to meet sleep duration recommendations to minimize the harmful association between short sleep duration and worse health indicators [54]. Thus, implementing health interventions becomes crucial in improving sleep duration and promoting physical activity among children and adolescents [55]. Furthermore, gaining a deeper understanding of the interrelationships between sleep, sedentary behavior, and physical activity is essential for enhancing overall health and well-being during adolescence [40]. In addition, improving mental well-being is important to promote physical activity and ideal sleep [43]. It is suggested that an intervention program that includes obesity-related health education, physical activity, and diet control is important to improve HQOL in the child, especially in boys [56]. Also, the HQOL seems to be benefited from an educational intervention program focused on physical activity and nutritional recommendations in children and adolescents with obesity [57].

Our study provides evidence consistent with others [17, 23, 24, 34], regarding the differences between boys and girls (β=0.24; *p* = 0.01). Males in our study showed a significant and positive association with healthy mediating factors and physical literacy itself. This can be explained by the fact that boys tend to prefer the type of class offered in the cultural context of the evaluated physical education, and they also appear to be more socially encouraged, although this is changing, to engage in physically active behavior [17, 23, 24, 34].

It is important to acknowledge some limitations of the present study. Firstly, the health-related quality of life (HQOL) and physical literacy components were based on self-reported measures, which may introduce potential biases and result in underestimation or overestimation of the results. Only a few components of physical literacy were assessed in the present study due to statistical limitations in identifying a reliable model. We suggest that others studies about physical literacy and SEM models being proposed to bring further discussions about the relationships between sleep and this complex component, as no SEM model is definitive [35]. On the other hand, it is worth noting some strengths of the study. This research represents one of the initial attempts in Latin America to propose a theoretical multivariate study utilizing Structural Equation Modeling (SEM) among adolescents. By employing this approach, the study ensures a mathematically appropriate and sustainable research design to explore the impact of sleep on various physical literacy components.

## Conclusion

There is an indirect relationship between ideal sleep duration and quality of life, and between both with physical literacy. These relationships occur even considering the negative influence of obesity in these SEM multiple relations. Furthermore, these relationships are more prominent among boys compared to girls. Therefore, strategies that are both nutritional and multicomponent are important for obesity control. In this regard, children and adolescents should also be educated about sleep habits. Sleep is a fundamental factor for children to develop their physical literacy and engage in physical education classes with greater enjoyment, pleasure, and satisfaction. Our study suggests that a child who sleeps adequately has a higher likelihood of being physically active, regardless of obesity. This, in turn, enhances overall quality of life across various domains, including physical well-being, psychological well-being, autonomy and relationships with parents, social support and peers, and the school environment, as assessed through a latent construct.

## Data Availability

Datasets used are available from the corresponding author on reasonable request by e-mail (vanilson.lemes@hotmail.com).
